# The moderating role of parent–child interaction in the relationship between maltreatment and psychological well-being among preschool children

**DOI:** 10.3389/fpsyg.2024.1471723

**Published:** 2025-01-06

**Authors:** Cheng Zhang, Chuican Huang, Chunhui Zhang, Weijia Wu, Zhenning Huang, Xue Xia, Sijia Liu, Chen Wang, Qing Luo, Lichun Fan

**Affiliations:** ^1^Hainan Women and Children’s Medical Center, School of Pediatrics, Hainan Academy of Medical Sciences, Hainan Medical University, Haikou, China; ^2^Department of Child Health Care, Hainan Women and Children’s Medical Center, Haikou, China; ^3^School of Public Health, Hainan Medical University, Haikou, China; ^4^National Center for Women and Children’s Health, Chinese Center for Disease Control and Prevention, Beijing, China

**Keywords:** child maltreatment, moderating analysis, preschool children, parent–child interaction, psychological well-being

## Abstract

**Objective:**

This study aimed to examine the relationship between maltreatment and psychological well-being among preschool children and explore parent–child interaction’s moderating role on this association.

**Methods:**

This study employed a cross-sectional design and multistage stratified cluster random sampling method. Between December 2022 and January 2023, 180 kindergartens in the Hainan province—encompassing 4,886 newly enrolled children as participants—were selected. All data were collected using an online platform, on which parents or caregivers reported their children’s history of abuse and completed the Strengths and Difficulties Questionnaire and Chinese Parent–Child Interaction Scale.

**Result:**

The reporting rate of child abuse in Hainan Province was 61.81%. Specifically, frequent physical abuse (odds ratio [*OR*] = 1.44, 95% confidence interval [*CI*] = 1.15–1.80), occasional physical neglect (*OR* = 1.75, 95% *CI* = 1.37–2.11), frequent physical neglect (*OR* = 1.57, 95% *CI* = 1.31–1.87), and frequent emotional abuse (*OR* = 1.57, 95% *CI* = 1.31–1.87) were associated with a higher overall rate of difficulties. Frequent physical abuse (*OR* = 1.33, 95% *CI* = 1.08–1.64) and frequent emotional abuse (*OR* = 1.36, 95% *CI* = 1.16–1.61) were correlated with a higher rate of prosocial problems. A significant interaction was observed between parent–child interaction and occasional physical neglect on psychological well-being (*OR* = 0.73, 95% *CI* = 0.54–0.98, *p* = 0.035).

**Conclusion:**

Preschool children who experience maltreatment exhibit an increased risk of developing psychological health issues, indicating a cumulative effect. Our study indicates that positive parent–child interaction mitigates occasional physical neglect’s negative impact on preschool children’s psychological well-being but does not exhibit a moderating effect on frequent physical neglect or other forms of maltreatment.

## 1 Introduction

Child maltreatment refers to actions by individuals who are obligated to raise, supervise, and exercise control over children that cause actual or potential harm to children’s health, survival, growth, development, and dignity (WHO, 1999). These actions encompass physical, emotional, and sexual abuse and physical and emotional neglect (Salum et al., 2016). Child maltreatment—as a form of child harm—maintains a persistently high prevalence and has emerged as a pressing public health concern, garnering attention from government officials and scholars worldwide. Its repercussions on children’s physical, social, and psychological well-being are intricate and far-reaching. Child maltreatment’s profound and destructive consequences affect individuals throughout their childhood, adolescence, and even into adulthood, shaping the course of their entire lives (Negriff et al., 2020; Lu et al., 2016). Comprehensive data regarding the prevalence of child maltreatment behavior reveals that approximately 50% of the global population of children aged 2–17 have experienced physical abuse (Hillis et al., 2016). Notably, younger children are more susceptible to being maltreated and exhibit a higher risk of suffering fatal consequences from abuse and neglect (Zeanah and Humphreys, 2018). The “Global Status Report on Preventing Violence Against Children 2020” indicates that nearly three-quarters of children aged 2–4 years frequently experience physical punishment and/or psychological violence from parents and caregivers. Furthermore, one-fifth of women and one in thirteen men report having experienced childhood sexual abuse (WHO, 2022). A meta-analysis in China revealed that the forms of child maltreatment are diverse (e.g., physical beating, verbal abuse, forced kneeling); moreover, among the 10 articles that fulfilled its inclusion criteria, the reported incidence of child maltreatment ranged from 30.5 to 72.3%, and a random effects model revealed the maltreatment rate to be 54.0% (95% *CI* = 42.0–67.0%) (Yang et al., 2014).

Most evidence linking child maltreatment and psychological health issues has been derived from retrospective reports obtained from adults participating in cross-sectional studies (Zhang et al., 2022; Dong et al., 2023). Numerous domestic and international studies have utilized the strengths and difficulties questionnaire (SDQ) to assess emotional and behavioral problems among children and adolescents. Research has demonstrated a significant association between child maltreatment and emotional and behavioral problems (Li et al., 2021; Zelviene et al., 2020). Studies have indicated that children who have—compared to those who have not—experienced maltreatment tend to exhibit lagging cognitive abilities (Platt et al., 2018). Simultaneously, this process can damage children’s brain structure, further affecting the development of their executive functions (Cowell et al., 2015). An analysis of 662 children and adolescents aged 10–16—measuring and assessing abuse history, memory, and the NEO Five-Factor personality traits—revealed a positive correlation between childhood abuse severity and memory impairment severity. Additionally, a significant negative correlation has been observed between child maltreatment and cognitive function scores (Scardera et al., 2023). Paulina Zelviene grouped adolescents who experienced childhood maltreatment using latent class analysis, finding that the severe maltreatment group (those who had experienced multiple types of maltreatment) reported higher levels of hyperactivity/inattention, showing a cumulative effect (Zelviene et al., 2020).

However, not all children who experience maltreatment develop psychological issue, suggesting that certain factors may play a protective or moderating role. In recent years, parent–child interaction has emerged as a key moderating factor of interest (Holz et al., 2020). Parent–child interaction refers to the psychological and emotional interplay between parents and their children—characterized by its familial, long-term, and affectionate nature (Cao et al., 2023). Parent–child interaction is a crucial indicator for assessing caregiving quality, with high-quality parent–child interaction being a determinant factor in children’s cognitive and psychosocial development (Rocha et al., 2020). Studies have suggested that parent–child interaction plays a positive promoting role in children’s psychological and behavioral development and also exerts beneficial effects on their cognitive functions (Rahkonen et al., 2014; Chavez et al., 2024), emotional and social competence (Grazuleviciene et al., 2017; Scherer et al., 2019; Poulain et al., 2019), and language abilities (Madigan et al., 2019; Justice et al., 2019; Garcia et al., 2019). Research has shown that even in cases where children experience abuse, a positive parent–child relationship can partially mitigate its adverse effects (Jiang et al., 2022). Therefore, parent–child interaction is not only a fundamental condition of child development but may also serve as a critical variable in moderating the impact of abuse on children’s psychological well-being. Given the intricate and varied nature of child maltreatment, along with the understanding that preschool-aged children are at a critical stage of psychological and emotional development, they are especially susceptible to adverse childhood experiences. Moreover, studies focusing on the mental health of preschool-aged children remain relatively scarce. To explore the relationships among parent–child interaction, child maltreatment, and children’s psychological well-being, we propose the following hypotheses: Do different types of maltreatment among preschool children exert cumulative effects on their psychological health issues? Does parent–child interaction moderate maltreatment’s negative psychological effects among preschool children?

## 2 Methods

### 2.1 Study design and ethics

This study is part of a cross-sectional survey based on an Early Childhood Development Intervention Strategy, comprising maltreatment, parent–child interaction, psychological health, and related factors. The survey was administered among newly enrolled children in kindergartens in Hainan Province from December 2022 to January 2023 and received ethical approval from the Ethics Committee of Hainan Women and Children’s Medical Center (Ethics Committee Approval Number: 2020002).

### 2.2 Participants

This study employed a stratified multistage cluster random sampling method to recruit newly enrolled children in Hainan Province in 2022. Children with foreign nationality or those attending special education schools were excluded. The specific sampling method is detailed in Xu J’s paper (Xu et al., 2023).

### 2.3 Sample size

The specific method utilized herein for sample size calculation is detailed in Xu J’s paper (Xu et al., 2023).

### 2.4 Measurements

An electronic questionnaire survey—encompassing self-designed questionnaires and internationally standardized questionnaires—was administered. All parents provided their informed consent electronically.

#### 2.4.1 Maltreatment history

Each parent provided their child’s maltreatment history—including physical, sexual, and emotional abuse and physical and emotional neglect—by answering the following questions:

Has the child ever been hit by an adult at home, including yourself, resulting in visible bruises or marks on the child?
Has the child ever experienced insufficient food or worn dirty or torn clothing?
Has the child ever been verbally insulted; called names, such as stupid, idiot, foolish, or worthless; or subjected to shouting?
Has anyone engaged in sexually related activities with the child or threatened to harm them if they refused?

Parents were asked to rate these questions on a 4-point scale (“1” = never, “2” = once or twice, “3” = sometimes, “4” = often). Subsequently, “never” was coded as “no,” “once or twice” as “occasionally,” and “sometimes” and “often” as “frequent.” A new variable—“cumulative abuse”—was created to represent the cumulative count of different types of abuse, including the following categories: no abuse, 1 type of abuse, 2 types of abuse, and 3 or 4 types of abuse. Another new variable—“global maltreatment”—was created to indicate the occurrence of any form of abuse. This scale’s reliability and validity have been validated in a community sample (Salum et al., 2016).

#### 2.4.2 SDQ

Children’s psychological well-being was assessed using the SDQ (parent version)—a rating scale used for screening the psychological well-being of 3–4-year-old children. The questionnaire comprises 25 items and five scales—namely, emotional symptoms, conduct problems, hyperactivity/inattention, peer problems, and prosocial problems. The prosocial problems subscale score reflects strengths, while the scores from the other four problem scales are summed to represent total difficulties. Parents rate their child’s behavior over the past 6 months on a scale ranging from 0 (not true) to 2 (certainly true). The total difficulties score is categorized into normal and abnormal groups, with a score greater than 16 indicating abnormality. Likewise, the prosocial problems subscale score is categorized into normal and abnormal groups, with a score less than 5 indicating abnormality. This scale has been validated in various regions of China, with Cronbach’s *α* coefficients ranging from 0.30 to 0.83 (Kou et al., 2007; Du et al., 2008). In this study, the questionnaire demonstrated acceptable reliability and validity, with Cronbach’s *α* coefficients ranging from 0.664 to 0.739.

#### 2.4.3 Chinese parent–child interaction scale (CPCIS)

Parent–child interaction was assessed using the CPCIS, which comprises 12 items representing parent–child interaction activities that may benefit child development, including activities related to learning, reading, entertainment, and interaction with the environment. Parents rate the frequency of their participation in these 12 parent–child interaction activities over the past month on a scale ranging from 0 (never) to 5 (very frequently). Higher scores indicate more frequent parent–child interaction activities. The CPCIS has been validated in China and demonstrates acceptable internal consistency (0.82) (Ip et al., 2018). In this study, the questionnaire demonstrated acceptable reliability and validity, with Cronbach’s α coefficients ranging from 0.774 to 0.923.

#### 2.4.4 Covariates

Child age, gender (girls, boys), *hukou* (rural, urban), only-child status (non-only child, only child), left-behind status (non-left-behind, left-behind), primary caregiver (parents, grandparents, others), parental education (middle school and below, high school/technical school, college/university, unknown), and family annual income (CNY0–CNY30,000, CNY30,000–CNY100,000, over CNY100,000, unknown) were reported by parents and used as covariates. Following definitions from various literature sources, this study defined left-behind children as rural children aged below 16, with one or both parents working away from home for more than 6 months (Zhou et al., 2020).

### 2.5 Quality control

This study employed scales with acceptable reliability and validity. We carried out a pilot survey involving 128 children who were chosen from two randomly selected kindergartens to verify their understanding of the questionnaire. Following their feedback, we implemented various changes to improve clarity. Nevertheless, the findings from this pilot survey were excluded from the main research study. Important survey indicators in the questionnaire were defined rigorously. Before the survey’s administration, uniform training was provided to the surveyors. Teachers provided feedback on issues encountered by parents when completing the questionnaire, and surveyors provided online guidance to address these concerns. Emphasize the anonymity and confidentiality of the questionnaire to make parents feel safe, encouraging them to answer sensitive questions more honestly. Relevant data collected were compiled into a basic database. We employed the list wise deletion method to exclude samples that did not meet the study criteria or lacked essential information. After assessing for non-response bias, the collected data were subsequently analyzed.

### 2.6 Statistical analysis

Descriptive statistics—including means, standard deviations, and proportions—were used to summarize the characteristics of all children included in this study. Binary logistic regression analysis was employed to assess the relationship between child abuse and psychological well-being (total difficulties, prosocial behavior) using odds ratios (*ORs*) and 95% confidence intervals (95% *CIs*); both unadjusted and adjusted analyses were conducted. Each model used different types of abuse, cumulative abuse, and interactions between different types of abuse and parent–child interaction as independent variables to investigate their relationships with psychological well-being (total difficulties and prosocial behavior). Statistical analyses were conducted using IBM SPSS Statistics 26 (SPSS Inc., Chicago, IL, United States), and graphs were created using GraphPad Prism 8.0 (GraphPad Software Corporation, La Jolla, CA, United States). Statistical significance was defined as *p* < 0.05.

## 3 Results

### 3.1 Participant characteristics

**Figure 1:**
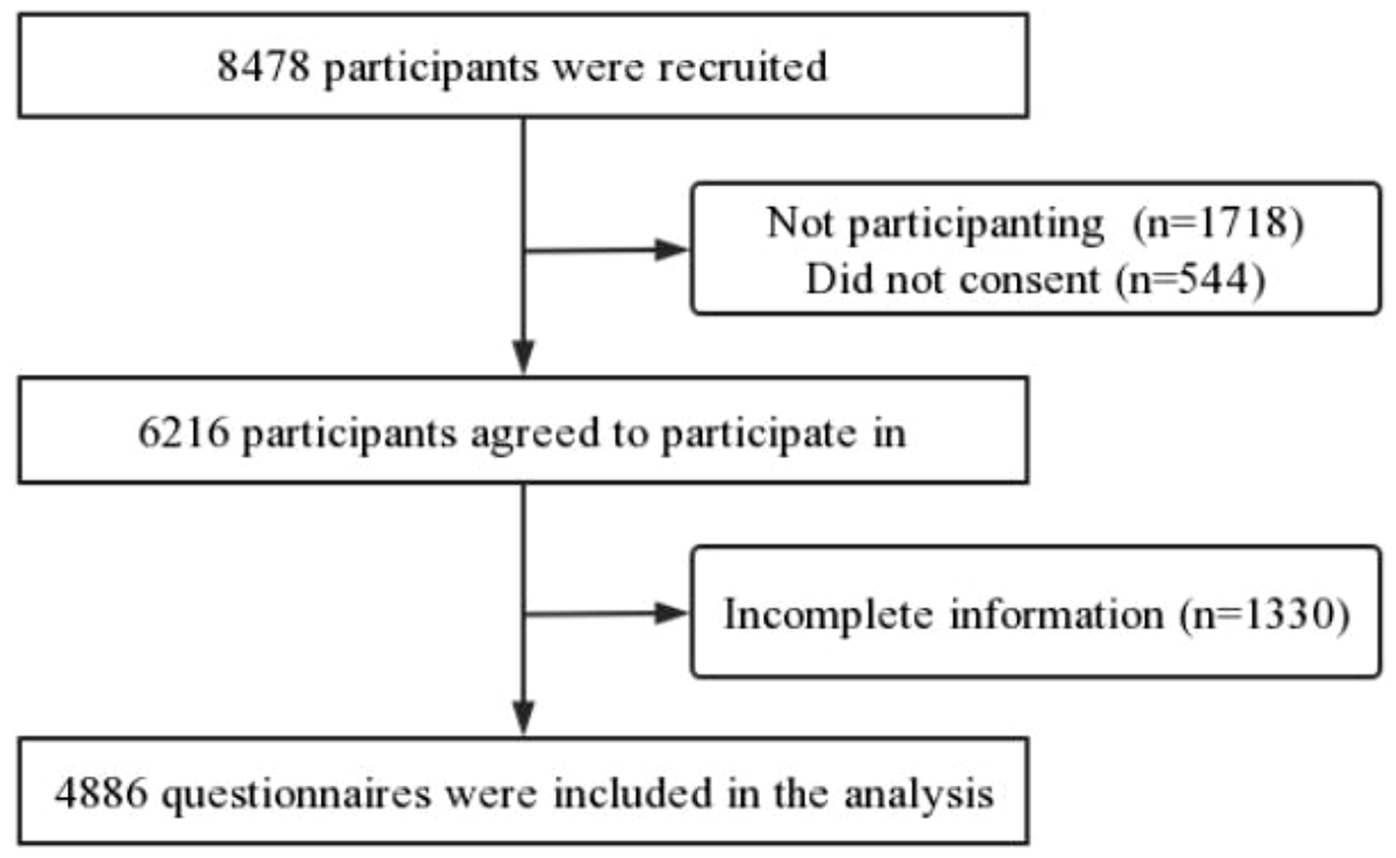
Flowchart of sample selection.

This study analyzed 4,886 valid questionnaires, with an effective response rate of 57.6% (Figure 1). The analysis revealed no statistically significant differences between respondents and non-respondents in terms of gender and age (Supplementary Table S1). Of the 4,886 children investigated (mean age = 3.68 ± 0.41 years), boys accounted for 54.86%. Table 1 presents the children’s characteristics. The reported child abuse prevalence in Hainan Province was 61.81%, with emotional abuse being the most common (49.92%), followed by physical abuse (26.73%), physical neglect (17.31%), and, finally, sexual abuse (3.27%). Regarding cumulative abuse, 1 type of abuse (34.14%), 2 types of abuse (20.61%), and 3 or 4 types of abuse (7.06%) were observed. The parent–child interaction level in Hainan Province exhibited a mean score of 29.27 ± 14.15. Among the children, 19.71% exhibited total difficulties, while 25.07% exhibited prosocial problems (Table 1).

**Table 1:** Descriptive characteristics of the participants.

Variables	N	Mean ± SD/proportion (%)
Age	4,886	3.68 ± 0.41
Gender		
Boys	2,680	54.85
Girls	2,206	45.15
Hukou*		
Rural	2,868	59.16
Urban	1,980	40.84
Siblings		
Only 1 child	1,660	33.97
None-only child	3,226	66.03
Left-behind children		
Yes	1,486	30.41
No	3,400	69.59
Primary caregiver		
Parents	4,072	83.34
Grandparents / maternal grandparents	773	15.82
Others	41	0.84
Paternal education level		
Junior high school and below	1,704	34.88
Senior high school/junior college	1,049	21.47
Undergraduate and above	1,969	40.30
Unknown	164	3.36
Maternal education level		
Junior high school and below	1,743	35.67
Senior high school/junior college	1,015	20.77
Undergraduate and above	1,990	40.73
Unknown	138	2.83
Family annual income (CNY)		
<30,000	801	16.39
30,000-100,000	996	20.38
>100,000	968	19.81
Unknown	2,121	43.41
Child maltreatment		
Physical abuse	1,306	26.73
Physical neglect	846	17.31
Emotional abuse	2,439	49.92
Sexual abuse	160	3.27
Cumulative maltreatment		
No	1,866	38.19
1 type	1,668	34.14
2 types	1,007	20.61
3 or 4 types	345	7.06
Global maltreatment	3,020	61.81
Parent–child interaction	4,886	29.27 ± 14.15
Total difficulties (>16)	963	19.71
Prosocial problems (<5)	1,225	25.07

^*^ Hukou type represents the registered place of residence for children (urban or rural areas).

### 3.2 Correlations between distinct forms of maltreatment, cumulative maltreatment, and psychological well-being

**Figure 2:**
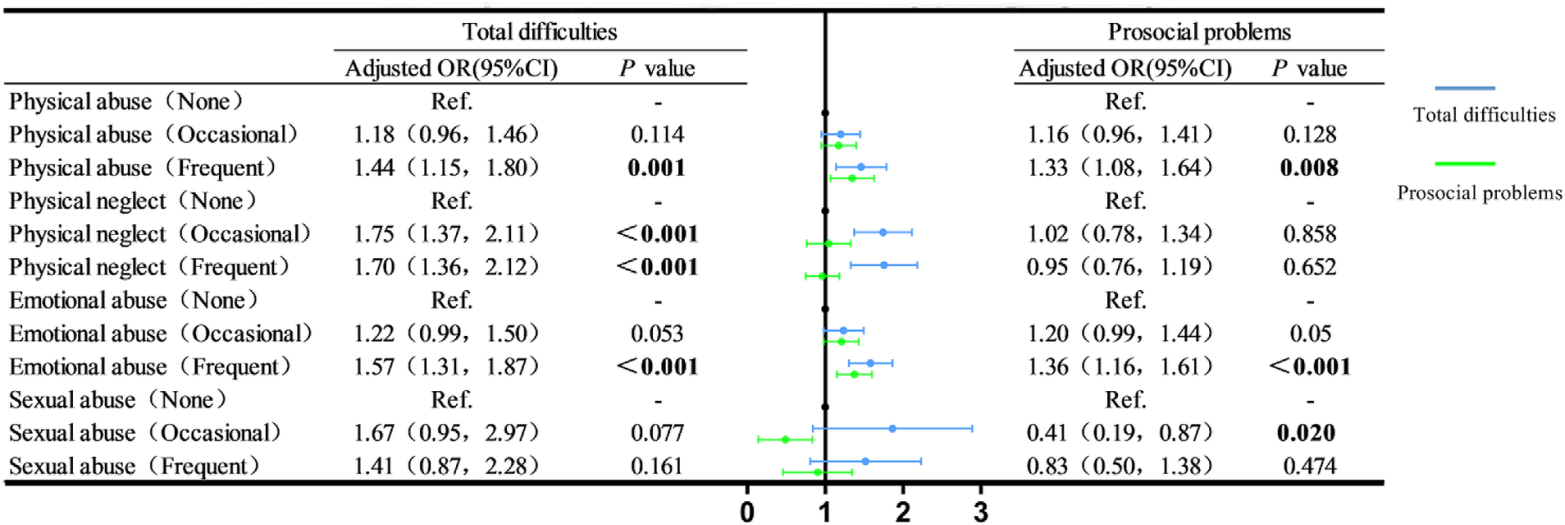
The relationship between different types of abuse and psychologically healthy. Adjusted for age, gender, urban–rural areas, siblings, primary caregiver, left-behind child, parental education level, family annual income and other types of abuse.

Figure 2 illustrates the association between different forms of maltreatment and psychological well-being. Children who had—compared to those who had not—experienced maltreatment exhibited the following results: Frequent physical abuse (*OR* = 1.44, 95% *CI* = 1.15–1.80), occasional physical neglect (*OR* = 1.75, 95% *CI* = 1.37–2.11), frequent physical neglect (*OR* = 1.57, 95% *CI* = 1.31–1.87), and frequent emotional abuse (*OR* = 1.57, 95% *CI* = 1.31–1.87) were associated with a higher total difficulty rate; frequent physical abuse (*OR* = 1.33, 95% *CI* = 1.08–1.64) and frequent emotional abuse (*OR* = 1.36, 95% *CI* = 1.16–1.61) were related to a higher rate of prosocial problems; and occasional sexual abuse (*OR* = 0.41, 95% *CI* = 0.19–0.87) was associated with a lower rate of prosocial problems.

**Figure 3:**
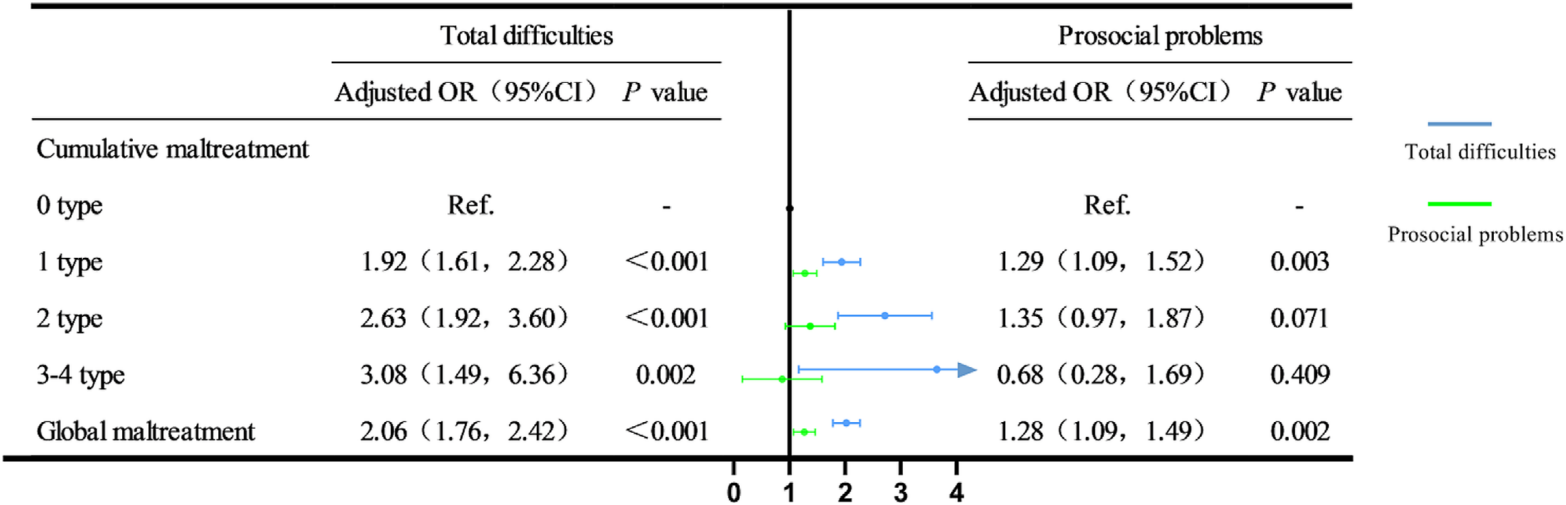
The relationship between different cumulative abuse and psychologically healthy. Adjusted for age, gender, urban–rural areas, siblings, primary caregiver, left-behind child, parental education level and family annual income.

Figure 3 presents the relationship between different cumulative maltreatment types and psychological well-being. This study demonstrates that as the number of cumulative maltreatment types increases, the likelihood of a higher overall difficulty problem rate also increases among children with—compared to those without—maltreatment. For one type of maltreatment (*OR* = 1.92, 95% *CI* = 1.61–2.28), two types of maltreatment (*OR* = 2.63, 95% *CI* = 1.92–3.60), and three or more types of maltreatment (*OR* = 3.08, 95% *CI* = 1.49–6.36) was associated with a higher rate of total difficulty. Global maltreatment was associated with higher rates of total difficulty (*OR* = 2.06, 95% *CI* = 1.76–2.42) and prosocial problems (*OR* = 1.28, 95% *CI* = 1.09–1.49).

### 3.3 Parent–child interaction’s moderating effect on various forms of maltreatment and their impact on psychological well-being

Table 2 presents the impact of physical neglect and the interaction term between physical neglect and parent–child interaction on psychological well-being. Compared to no history of physical neglect, occasional physical neglect (*OR* = 1.62, 95% *CI* = 1.22–2.16) and frequent physical neglect (*OR* = 2.10, 95% *CI* = 1.68–2.63) were associated with a higher prevalence of total difficulties. Higher scores in parent–child interaction exhibited a correlation with a lower rate of total difficulties (*OR* = 0.98, 95% *CI* = 0.98–0.99). Occasional physical neglect and its interaction with parent–child interaction significantly impacted child psychological well-being (*OR* = 0.73, 95% *CI* = 0.54–0.98), while frequent physical neglect and its interaction with parent–child interaction did not significantly impact child psychological well-being (*OR* = 1.14, 95% *CI* = 0.92–1.41). Further, occasional physical abuse (*OR* = 0.86, 95% *CI* = 0.69–1.08) and frequent physical abuse (*OR* = 1.08, 95% *CI* = 0.87–1.34) did not significantly impact child psychological well-being when interacting with parent–child interaction. Likewise, occasional emotional abuse (*OR* = 0.92, 95% *CI* = 0.75–1.13) and frequent emotional abuse (*OR* = 1.06, 95% *CI* = 0.89–1.26) did not significantly impact children’s psychological well-being in interaction with parent–child interaction. Finally, occasional sexual abuse (*OR* = 0.99, 95% *CI* = 0.96–1.04) also did not significantly impact child psychological well-being in interaction with parent–child interaction (Supplementary Tables S2–S4).

**Table 2:** The effect of physical neglect and parent–child interaction on psychologically health.

	Total difficulties			Prosocial problems		
	*β*	OR (95% CI)	*p* value	*β*	OR (95% CI)	*P* value
Age	−0.10	0.90 (0.76, 1.08)	0.255	−0.51	0.60 (0.51, 0.71)	**<0.001**
Gender						
Boys	Ref.	Ref.	–	Ref.	Ref.	–
Girls	−0.03	0.97 (0.84, 1.13)	0.706	−0.32	0.73 (0.63, 0.83)	**<0.001**
Hukou*						
Rural	Ref.	Ref.	–	Ref.	Ref.	–
Urban	−0.11	0.90 (0.73, 1.10)	0.290	−0.20	0.82 (0.68, 0.99)	**0.035**
Siblings						
Only 1 child	Ref.	Ref.	–	Ref.	Ref.	–
None-only child	−0.06	0.94 (0.80, 1.11)	0.470	−0.03	0.97 (0.83, 1.12)	0.658
Left-behind children						
Yes	Ref.	Ref.	–	Ref.	Ref.	–
No	−0.06	0.95 (0.79, 1.13)	0.539	0.23	1.26 (1.06, 1.50)	**0.008**
Primary caregiver						
Parents	Ref.	Ref.	–	Ref.	Ref.	–
Grandparents / maternal grandparents	−0.08	0.93 (0.75, 1.15)	0.484	0.12	1.13 (0.94, 1.36)	0.209
Others	−0.21	0.81 (0.33, 1.97)	0.638	0.34	1.40 (0.70, 2.80)	0.346
Paternal education level						
Junior high school and below	Ref.	Ref.	–	Ref.	Ref.	–
Senior high school/junior college	0.02	1.02 (0.83, 1.26)	0.852	−0.25	0.78 (0.64, 0.96)	**0.021**
Undergraduate and above	−0.21	0.81 (0.63, 1.04)	0.092	0.04	1.04 (0.83, 1.31)	0.718
Unknown	0.70	2.02 (1.18, 3.48)	**0.011**	−0.06	0.94 (0.54, 1.63)	0.832
Maternal education level						
Junior high school and below	Ref.	Ref.	–	Ref.	Ref.	–
Senior high school/junior college	−0.10	0.90 (0.73, 1.12)	0.350	0.02	1.02 (0.83, 1.25)	0.871
Undergraduate and above	−0.19	0.83 (0.65, 1.06)	0.128	−0.06	0.94 (0.75, 1.19)	0.612
Unknown	−0.47	0.62 (0.34, 1.16)	0.135	0.17	1.19 (0.66, 2.16)	0.566
Family annual income (CNY)						
<30,000	Ref.	Ref.	–	Ref.	Ref.	–
30,000-100,000	0.01	1.01 (0.80, 1.27)	0.968	0.06	1.06 (0.85, 1.33)	0.597
>100,000	−0.25	0.78 (0.58, 1.04)	0.084	−0.05	0.95 (0.73, 1.24)	0.703
Unknown	−0.12	0.88 (0.72, 1.08)	0.229	0.01	1.00 (0.82, 1.22)	0.975
Physical neglect (None)	Ref.	Ref.	–	Ref.	Ref.	–
Physical neglect (Occasional)	0.48	1.62 (1.22, 2.16)	**0.001**	0.04	1.04 (0.78, 1.39)	0.803
Physical neglect (Frequent)	0.74	2.10 (1.68, 2.63)	**<0.001**	−0.03	0.97 (0.75, 1.26)	0.824
Parent–child interaction	−0.02	0.98 (0.98, 0.99)	**<0.001**	−0.04	0.96 (0.95, 0.97)	**<0.001**
Physical neglect (None) × Parent–child interaction	Ref.	Ref.	–	Ref.	Ref.	–
Physical neglect (Occasional) × Parent–child interaction	−0.32	0.73 (0.54, 0.98)	**0.035**	0.06	1.06 (0.79, 1.43)	0.707
Physical neglect (Frequent) × Parent–child interaction	0.13	1.14 (0.92, 1.41)	0.231	−0.07	0.94 (0.73, 1.20)	0.597

CNY: Chinese Yuan. Adjusted for age, gender, urban–rural areas, siblings, primary caregiver, left-behind child, parental education level and family annual income. Bold values indicates *p* < 0.05.

## 4 Discussion

Child maltreatment—a highly complex phenomenon involving various forms—can occur in any setting where children grow up, both within and outside the family (Yang et al., 2014). In China’s traditional cultural context, deep-rooted cultural beliefs—such as “spare the rod, spoil the child” and “physical discipline is a form of love”—have contributed to the prevalence of childhood maltreatment (Qiao and Chan, 2005). Owing to historical inconsistencies in study populations’ definitions, measurements, and characteristics in prior research, particularly concerning the concealed nature of child abuse within the family, such instances have frequently remained undisclosed or underreported. Reporting rates of child abuse range widely across China from 3.5% to over 90% (Sun, 2021; Shan et al., 2019). This study reveals a child maltreatment prevalence rate of 61.81% among preschool children—as reported by parents in Hainan Province—slightly higher than the 58.6% reported by a study conducted in Shanghai (Wang et al., 2022). Further, this study reveals that the reporting rate for child physical neglect is 17.31%, surpassing that of Shanghai (6.98%), Australia (4.1%) and Saudi Arabia (5.5%) (Mathews et al., 2023; AlFarhan et al., 2022). The reporting rates for the other three forms of abuse exhibit minimal differences. The problem corresponding to physical neglect—whether the child has experienced insufficient access to food or has worn dirty or torn clothing—is closely related to their family’s economic situation. The higher reporting rate of physical neglect observed in this study is primarily attributable to the higher per capita income in Shanghai and substantial differences in the population’s demographic characteristics. Additionally, Hainan Province’s unique geographical location—influenced by high temperatures—may result in a different interpretation of the term “dirty clothing” compared to other regions. This study finds that the reporting rate for sexual abuse is the lowest among the four types of abuse (3.27%), which aligns closely with findings from other regions in China (Shan et al., 2019). Further, the reporting rates for physical and sexual abuse observed in this study are greater than other countries and lower than the global average, respectively (Yu et al., 2024; Langevin et al., 2022). This is attributable to the conservative attitude toward “sex” within the traditional sociocultural context in China, which may introduce some reporting bias in cases of sexual abuse.

Consistent with prior studies (Negriff et al., 2020; Lee and Chen, 2017; LeMoult et al., 2020), this study finds that frequent instances of physical abuse, physical neglect, and emotional abuse adversely impact children’s mental health—specifically, emotional symptoms, conduct problems, hyperactivity/inattention, and peer relationship problems. Moreover, frequent physical and emotional abuse negatively impact children’s psychosocial well-being (prosocial problems). Our findings robustly support the notion of child maltreatment detrimentally impacting early psychological well-being. However, our study also yielded an unexpected result: Occasional sexual abuse was associated with a lower incidence of prosocial problems. Sexual abuse remains a sensitive topic in Chinese society, and hence, we only conducted a basic analysis of sexual abuse. Additionally, within the Chinese cultural context, we explored in-depth why parent–child interaction mitigates physical neglect’s impact on children’s psychological well-being but does not affect other forms of abuse (such as emotional or physical abuse). This finding contrasts our expectations as well as some prior studies. Considering that sexuality-related issues are often particularly prone to reporting bias, it may be related to the relatively low reporting rate of sexual abuse within the sample (Ma, 2018; Sanjeevi et al., 2018). This study’s results reveal that a greater number of types of child maltreatment are associated with increased total difficulties, demonstrating a cumulative effect. Although children with multiple experiences may age over time, the potential for cumulative effects exists insofar as children have experienced more than one type of child maltreatment (Turner et al., 2010). Furthermore, the negative impact on children is more pronounced the earlier these experiences occur (Heim et al., 2013).

This study reveals a significant interaction between occasional physical neglect and parent–child interaction vis-à-vis total difficulties among children (Table 2). This finding indicates that positive parent–child interaction buffers occasional physical neglect’s negative impact on total difficulties, which aligns with attachment theory and further supports the view that positive parent–child relationships mitigate maltreatment’s adverse effects on children’s early psychological well-being (Steele and McKinney, 2020; Kim-Spoon et al., 2012). However, we do not observe this interaction in the case of frequent physical neglect possibly because frequent physical neglect severely impacts the functioning of the hypothalamic–pituitary–adrenal axis, precipitating irreversible brain damage and, consequently, the disappearance of parent–child interaction’s buffering effect (Burnette et al., 2012). Furthermore, we find that the interaction of parent–child interaction with abuse manifests in the context of physical neglect but not in the other three types of abuse (Supplementary Tables S2–S4)—possibly because the questions assessing whether children experienced “physical neglect” in the questionnaire tend to be more related to economic factors. In the future, we will explore the combined influence of family socioeconomic status and parent–child interaction on children’s psychological well-being. Irrespective of whether children have experienced abuse, positive parent–child interaction exerts a protective effect on their psychological well-being, highlighting the crucial roles of abuse and parent–child interaction in children’s psychological development. Previous intervention measures have primarily focused on mitigating child abuse. However, future interventions should also emphasize developing positive parent–child relationships.

### 4.1 Limitations and strengths

This cross-sectional study was conducted in China’s only tropical island province, where research on preschool children as a special group is relatively scarce in this field. Focusing on this group can fill several research gaps and provide further data support. However, when interpreting this study’s results, several limitations should be considered. First, due to the low response rate and limited information collected from non-respondents, a comprehensive comparison between the two groups was not possible, and the presence of non-response bias cannot be ruled out. Second, this study adopted a cross-sectional design, rendering it challenging to establish causal relationships between preschool child maltreatment and psychological well-being. Future research should utilize longitudinal data to examine causality. Third, the data employed in this study were reported by parents, which may have resulted in underreporting owing to the concealment or omission of abuse. This issue—possibly introducing information bias—may have resulted in an underestimation of the prevalence of maltreatment. Final, categorizing all forms of child abuse into a single category may have obscured gender differences.

## 5 Conclusion

This study revealed the prevalence of abuse among preschool children. Despite the adverse effects of emotional and physical abuse and physical neglect on children’s psychological well-being, child abuse is evidently associated with overall difficulties and also exhibits a cumulative effect. However, positive parent–child interaction may ameliorate occasional physical neglect’s negative impact on psychological well-being. These findings emphasize the importance for policymakers and caregivers to prioritize and cultivate positive, non-abusive parent–child relationships, which are conducive to school-age children’s psychological well-being.

## Data Availability

The raw data supporting the conclusions of this article will be made available by the authors, without undue reservation.
