# Supplementary material for: The moderating role of parent–child interaction in the relationship between maltreatment and psychological well-being among preschool children
Source: Front Psychol. 2025 Jan 6;15:1471723. doi: 10.3389/fpsyg.2024.1471723 (PMC11743930; doi:10.3389/fpsyg.2024.1471723)
Supplement: Supplementary file 1 [file Table_1.DOCX]

**Supplementary Information**

| **Table S1 Demographic data of respondents and non-respondents** | | | | | |
| --- | --- | --- | --- | --- | --- |
| **Variable** | **Total**  **(N = 8478)** | **Responder**  **(n = 4886)** | **Non-responder (n = 3592)** | ***t/χ^2^*** | ***P* value** |
| **Age** | 8478 | 3.68 ± 0.41 | 3.70 ± 2.39 | -0.574 | 0.556 |
| **Gender** |  |  |  |  |  |
| Boys | 4651(54.86) | 2677(54.79) | 1974(54.96) | 0.023 | 0.879 |
| Girls | 3827(45.14) | 2209(45.21) | 1618(45.04) |  |  |

| **Table S2 The effect of sexual abuse and parent-child interaction on psychologically health** | | | | | | | |
| --- | --- | --- | --- | --- | --- | --- | --- |
| **Variable** | **Total difficulties** | | |  | **Prosocial problems** | | |
|  | *β* | *OR* (95%*CI*) | *P* value |  | *β* | *OR* (95%*CI*) | *P* value |
| **Age** | -0.09 | 0.98(0.85, 1.14) | 0.288 |  | -0.50 | 0.61(0.52, 0.71) | **＜0.001** |
| **Gender** |  |  |  |  |  |  |  |
| Boys | Ref. | Ref. | - |  | Ref. | Ref. | - |
| Girls | -0.02 | 0.91(0.77, 1.08) | 0.800 |  | -0.32 | 0.73(0.63, 0.83) | **＜0.001** |
| **Hukou*** |  |  |  |  |  |  |  |
| Rural | Ref. | Ref. | - |  | Ref. | Ref. | - |
| Urban | -0.14 | 0.87(0.71, 1.07) | 0.185 |  | -0.21 | 0.81(0.67, 0.98) | 0.031 |
| **Siblings** |  |  |  |  |  |  |  |
| Only 1 child | Ref. | Ref. | - |  | Ref. | Ref. | - |
| None-only child | -0.05 | 0.96(0.81, 1.12) | 0.575 |  | -0.03 | 0.97(0.83, 1.13) | 0.696 |
| **Left-behind children** |  |  |  |  |  |  |  |
| Yes | Ref. | Ref. | - |  | Ref. | Ref. | - |
| No | -0.04 | 0.96(0.80, 1.15) | 0.667 |  | 0.24 | 1.27(1.06, 1.50) | **0.008** |
| **Primary caregiver** |  |  |  |  |  |  |  |
| Parents | Ref. | Ref. | - |  | Ref. | Ref. | - |
| Grandparents / maternal grandparents | -0.09 | 0.91(0.74, 1.13) | 0.393 |  | 0.12 | 1.13(0.94, 1.36) | 0.202 |
| Others | -0.03 | 0.74(0.30, 1.81) | 0.511 |  | 0.37 | 1.45(0.72, 2.92) | 0.296 |
| **Paternal education level** |  |  |  |  |  |  |  |
| Junior high school and below | Ref. | Ref. | - |  | Ref. | Ref. | - |
| Senior high school/junior college | -0.01 | 0.99(0.81, 1.22) | 0.945 |  | -0.25 | 0.78(0.63, 0.96) | **0.020** |
| Undergraduate and above | -0.23 | 0.79(0.62, 1.01) | 0.062 |  | 0.05 | 1.05(0.83, 1.32) | 0.681 |
| Unknown | 0.64 | 1.89(1.09, 3.26) | **0.023** |  | -0.07 | 0.93(0.54, 1.61) | 0.797 |
| **Maternal education level** |  |  |  |  |  |  |  |
| Junior high school and below | Ref. | Ref. | - |  | Ref. | Ref. | - |
| Senior high school/junior college | -0.15 | 0.86(0.69, 1.06) | 0.153 |  | 0.01 | 1.01(0.82, 1.24) | 0.923 |
| Undergraduate and above | -0.27 | 0.76(0.60, 0.98) | **0.031** |  | -0.06 | 0.95(0.75, 1.19) | 0.635 |
| Unknown | -0.54 | 0.58(0.31, 1.09) | 0.093 |  | 0.19 | 1.21(0.67, 2.20) | 0.528 |
| **Family annual income (CNY)** |  |  |  |  |  |  |  |
| ＜30,000 | Ref. | Ref. | - |  | Ref. | Ref. | - |
| 30,000-100,000 | -0.05 | 0.95(0.76, 1.20) | 0.678 |  | 0.05 | 1.05(0.84, 1.32) | 0.667 |
| ＞100,000 | -0.32 | 0.73(0.55, 0.97) | **0.029** |  | -0.06 | 0.94(0.72, 1.22) | 0.636 |
| Unknown | -0.18 | 0.84(0.69, 1.02) | 0.079 |  | -0.01 | 0.99(0.81, 1.21) | 0.949 |
| Sexual abuse (None) | Ref. | Ref. | - |  | Ref. | Ref. | - |
| Sexual abuse (Occasional) | 0.73 | 2.08(0.66, 6.57) | 0.214 |  | -2.07 | 0.13(0.03, 0.60) | **0.009** |
| Sexual abuse (Frequent) | -0.63 | 0.53(0.17, 1.69) | 0.284 |  | -0.15 | 0.86(0.27, 2.77) | 0.804 |
| Parent-child interaction | -0.02 | 0.98(0.97, 0.99) | **＜0.001** |  | -0.04 | 0.96(0.95, 0.96) | **＜0.001** |
| Sexual abuse (None)×Parent-child interaction | Ref. | Ref. | - |  | Ref. | Ref. | - |
| Sexual abuse (Occasional)×Parent-child interaction | 0.01 | 0.99(0.96, 1.04) | 0.932 |  | 0.05 | 1.05(1.01, 1.11) | **0.047** |
| Sexual abuse (Frequent)×Parent-child interaction | 0.04 | 1.04(1.01, 1.07) | **0.040** |  | 0.01 | 1.01(0.97, 1.05) | 0.771 |

Adjusted for age, gender, urban-rural areas, siblings, primary caregiver, left-behind child, parental education level and family annual income.

| **Table S3 The effect of emotional abuse and parent-child interaction on psychologically health** | | | | | | | |
| --- | --- | --- | --- | --- | --- | --- | --- |
| **Variable** | **Total difficulties** | | |  | **Prosocial problems** | | |
|  | *β* | *OR* (95%*CI*) | *P* value |  | *β* | *OR* (95%*CI*) | *P* value |
| **Age** | -0.09 | 0.91(0.77, 1.08) | 0.292 |  | -0.51 | 0.60(0.51, 0.71) | **＜0.001** |
| **Gender** |  |  |  |  |  |  |  |
| Boys | Ref. | Ref. | - |  | Ref. | Ref. | - |
| Girls | -0.01 | 0.99(0.86, 1.15) | 0.903 |  | -0.32 | 0.73(0.63, 0.84) | **＜0.001** |
| **Hukou*** |  |  |  |  |  |  |  |
| Rural | Ref. | Ref. | - |  | Ref. | Ref. | - |
| Urban | -0.14 | 0.87(0.71, 1.06) | 0.171 |  | -0.21 | 0.81(0.67, 0.98) | **0.030** |
| **Siblings** |  |  |  |  |  |  |  |
| Only 1 child | Ref. | Ref. | - |  | Ref. | Ref. | - |
| None-only child | -0.06 | 0.94(0.80, 1.11) | 0.460 |  | -0.04 | 0.96(0.83, 1.12) | 0.595 |
| **Left-behind children** |  |  |  |  |  |  |  |
| Yes | Ref. | Ref. | - |  | Ref. | Ref. | - |
| No | -0.01 | 0.99(0.83, 1.18) | 0.900 |  | 0.25 | 1.28(1.08, 1.52) | **0.005** |
| **Primary caregiver** |  |  |  |  |  |  |  |
| Parents | Ref. | Ref. | - |  | Ref. | Ref. | - |
| Grandparents / maternal grandparents | -0.06 | 0.94(0.76, 1.16) | 0.562 |  | 0.13 | 1.13(0.94, 1.37) | 0.188 |
| Others | -0.23 | 0.80(0.33, 1.95) | 0.619 |  | 0.37 | 1.44(0.72, 2.91) | 0.306 |
| **Paternal education level** |  |  |  |  |  |  |  |
| Junior high school and below | Ref. | Ref. | - |  | Ref. | Ref. | - |
| Senior high school/junior college | 0.01 | 1.01(0.81, 1.24) | 0.962 |  | -0.24 | 0.79(0.64, 0.97) | **0.023** |
| Undergraduate and above | -0.20 | 0.82(0.64, 1.05) | 0.110 |  | 0.06 | 1.06(0.84, 1.33) | 0.635 |
| Unknown | 0.57 | 1.77(1.03, 3.05) | **0.040** |  | -0.07 | 0.93(0.54, 1.62) | 0.808 |
| **Maternal education level** |  |  |  |  |  |  |  |
| Junior high school and below | Ref. | Ref. | - |  | Ref. | Ref. | - |
| Senior high school/junior college | -0.14 | 0.87(0.70, 1.07) | 0.188 |  | 0.02 | 1.02(0.83, 1.25) | 0.855 |
| Undergraduate and above | -0.28 | 0.76(0.59, 0.97) | **0.025** |  | -0.07 | 0.94(0.74, 1.18) | 0.579 |
| Unknown | -0.38 | 0.68(0.37, 1.27) | 0.228 |  | 0.23 | 1.26(0.69, 2.28) | 0.451 |
| **Family annual income (CNY)** |  |  |  |  |  |  |  |
| ＜30,000 | Ref. | Ref. | - |  | Ref. | Ref. | - |
| 30,000-100,000 | -0.03 | 0.97(0.77, 1.22) | 0.772 |  | 0.07 | 1.07(0.86, 1.34) | 0.541 |
| ＞100,000 | -0.31 | 0.74(0.55, 0.98) | **0.036** |  | -0.04 | 0.96(0.74, 1.25) | 0.784 |
| Unknown | -0.18 | 0.84(0.69, 1.02) | 0.083 |  | 0.01 | 1.01(0.83, 1.23) | 0.930 |
| Emotional abuse (None) | Ref. | Ref. | - |  | Ref. | Ref. | - |
| Emotional abuse (Occasional) | 0.22 | 1.25(1.02, 1.52) | **0.034** |  | 0.11 | 1.11(0.91, 1.35) | 0.276 |
| Emotional abuse (Frequent) | 0.58 | 1.79(1.52, 2.13) | **＜0.001** |  | 0.26 | 1.30(1.10, 1.53) | **0.002** |
| Parent-child interaction | -0.02 | 0.98(0.97, 0.99) | **＜0.001** |  | -0.04 | 0.96(0.95, 0.97) | **＜0.001** |
| Emotional abuse (None)×Parent-child interaction | Ref. | Ref. | - |  | Ref. | Ref. | - |
| Emotional abuse (Occasional)×Parent-child interaction | -0.09 | 0.92(0.75, 1.13) | 0.427 |  | -0.16 | 0.85(0.70, 1.05) | 0.124 |
| Emotional abuse (Frequent)×Parent-child interaction | 0.06 | 1.06(0.89, 1.26) | 0.501 |  | 0.02 | 1.02(0.86, 1.21) | 0.793 |

Adjusted for age, gender, urban-rural areas, siblings, primary caregiver, left-behind child, parental education level and family annual income.

| **Table S4 The effect of physical abuse and parent-child interaction on psychologically health** | | | | | | | |
| --- | --- | --- | --- | --- | --- | --- | --- |
| **Variable** | **Total difficulties** | | |  | **Prosocial problems** | | |
|  | *β* | *OR* (95%*CI*) | *P* value |  | *β* | *OR* (95%*CI*) | *P* value |
| **Age** | -0.08 | 0.92(0.78, 1.10) | 0.362 |  | -0.51 | 0.60(0.51, 0.71) | **＜0.001** |
| **Gender** |  |  |  |  |  |  |  |
| Boys |  |  |  |  |  |  |  |
| Girls | -0.001 | 0.99(0.86, 1.16) | 0.994 |  | -0.31 | 0.73(0.64, 0.84) | **＜0.001** |
| **Hukou*** |  |  |  |  |  |  |  |
| Rural | Ref. | Ref. | - |  | Ref. | Ref. | - |
| Urban | -0.13 | 0.88(0.72, 1.08) | 0.227 |  | -0.20 | 0.82(0.68, 0.99) | **0.042** |
| **Siblings** |  |  |  |  |  |  |  |
| Only 1 child | Ref. | Ref. | - |  | Ref. | Ref. | - |
| None-only child | -0.06 | 0.94(0.80, 1.10) | 0.441 |  | -0.04 | 0.96(0.83, 1.11) | 0.578 |
| **Left-behind children** |  |  |  |  |  |  |  |
| Yes | Ref. | Ref. | - |  | Ref. | Ref. | - |
| No | -0.04 | 0.96(0.80, 1.15) | 0.634 |  | 0.23 | 1.26(1.06, 1.49) | **0.010** |
| **Primary caregiver** |  |  |  |  |  |  |  |
| Parents | Ref. | Ref. | - |  | Ref. | Ref. | - |
| Grandparents / maternal grandparents | -0.06 | 0.94(0.76, 1.16) | 0.561 |  | 0.13 | 1.14(0.95, 1.37) | 0.172 |
| Others | -0.23 | 0.79(0.33, 1.94) | 0.612 |  | 0.36 | 1.43(0.71, 2.87) | 0.318 |
| **Paternal education level** |  |  |  |  |  |  |  |
| Junior high school and below | Ref. | Ref. | - |  | Ref. | Ref. | - |
| Senior high school/junior college | 0.02 | 1.02(0.83, 1.26) | 0.842 |  | -0.24 | 0.79(0.64, 0.97) | **0.026** |
| Undergraduate and above | -0.21 | 0.81(0.63, 1.04) | 0.092 |  | 0.05 | 1.06(0.84, 1.33) | 0.644 |
| Unknown | 0.66 | 1.93(1.12, 3.33) | **0.019** |  | 0.04 | 0.96(0.56, 1.67) | 0.894 |
| **Maternal education level** |  |  |  |  |  |  |  |
| Junior high school and below | Ref. | Ref. | - |  | Ref. | Ref. | - |
| Senior high school/junior college | -0.14 | 0.87(0.70, 1.07) | 0.190 |  | 0.02 | 1.02(0.83, 1.25) | 0.838 |
| Undergraduate and above | -0.25 | 0.78(0.61, 0.99) | **0.045** |  | -0.05 | 0.99(0.75, 1.19) | 0.647 |
| Unknown | -0.53 | 0.59(0.32, 1.11) | 0.099 |  | 0.17 | 1.19(0.66, 2.15) | 0.570 |
| **Family annual income (CNY)** |  |  |  |  |  |  |  |
| ＜30,000 | Ref. | Ref. | - |  | Ref. | Ref. | - |
| 30,000-100,000 | -0.05 | 0.95(0.76, 1.20) | 0.666 |  | 0.06 | 1.06(0.85, 1.33) | 0.610 |
| ＞100,000 | -0.32 | 0.73(0.55, 0.97) | 0.031 |  | -0.05 | 0.96(0.74, 1.24) | 0.736 |
| Unknown | -0.17 | 0.84(0.69, 1.03) | 0.096 |  | 0.01 | 1.01(0.83, 1.22) | 0.954 |
| Physical abuse (None) | Ref. | Ref. | - |  | Ref. | Ref. | - |
| Physical abuse (Occasional) | 0.24 | 1.27(1.03, 1.57) | **0.026** |  | 0.17 | 1.19(0.97, 1.45) | 0.091 |
| Physical abuse (Frequent) | 0.61 | 1.85(1.49, 2.29) | **＜0.001** |  | 0.30 | 1.35(1.08, 1.69) | **0.008** |
| Parent-child interaction | -0.02 | 0.98(0.98, 0.99) | **＜0.001** |  | -0.04 | 0.96(0.95, 0.96) | **＜0.001** |
| Physical abuse (None)×Parent-child interaction | Ref. | Ref. | - |  | Ref. | Ref. | - |
| Physical abuse (Occasional)×Parent-child interaction | -0.15 | 0.86(0.69, 1.08) | 0.197 |  | 0.04 | 1.04(0.84, 1.30) | 0.719 |
| Physical abuse (Frequent)×Parent-child interaction | 0.08 | 1.08(0.87, 1.34) | 0.488 |  | 0.06 | 1.06(0.85, 1.32) | 0.614 |

Adjusted for age, gender, urban-rural areas, siblings, primary caregiver, left-behind child, parental education level and family annual income.
